# Disruption of IL-33 Signaling Limits Early CD8+ T Cell Effector Function Leading to Exhaustion in Murine Hemophagocytic Lymphohistiocytosis

**DOI:** 10.3389/fimmu.2018.02642

**Published:** 2018-11-20

**Authors:** Julia E. Rood, Thomas N. Burn, Vanessa Neal, Niansheng Chu, Edward M. Behrens

**Affiliations:** ^1^Division of Rheumatology, Children's Hospital of Philadelphia, Philadelphia, PA, United States; ^2^Institute for Immunology, Perelman School of Medicine at the University of Pennsylvania, Philadelphia, PA, United States

**Keywords:** interleukin-33, ST2, hemophagocytic lymphohistiocytosis, CD8+ T cells, exhaustion, perforin, LCMV

## Abstract

Danger signals mediated through ST2, the interleukin-33 (IL-33) receptor, amplify CD8^+^ T cell-mediated inflammation in the murine model of familial hemophagocytic lymphohistiocytosis type 2 (FHL2), and blockade of ST2 provides a potential therapeutic strategy in this disease. However, the long-term effects of disrupting IL-33/ST2 signaling on the CD8^+^ T cell compartment are unknown. Here, we examined the evolution of the T cell response in murine FHL type 2 in the absence of ST2 signaling and found that CD8^+^ T cells gradually undergo exhaustion, similar to a related nonfatal FHL model. ST2 inhibition indirectly promotes CD8^+^ T cell exhaustion, and in contrast to other forms of FHL, reversal of exhaustion does not affect mortality. Disruption of IL-33 signaling exerts a more significant impact on the CD8^+^ T cell compartment early in the course of disease by intrinsically limiting CD8^+^ T cell proliferative and cytokine production capacity. Our data thus suggest that while ST2 blockade ultimately enables the development of CD8^+^ T cell exhaustion in late-stage murine FHL2, exhaustion is merely an effect, rather than the cause, of extended survival in these mice. The acute impact of ST2 inhibition on both the quantity and quality of the effector CD8^+^ T cell response more likely underlies the protective benefits of this treatment. This study provides evidence that redefines the relationship between CD8^+^ T cell exhaustion and mortality in murine FHL and supports the therapeutic use of ST2 blockade during the acute stage of disease.

## Introduction

Hemophagocytic lymphohistiocytosis is a disease of dysregulated hyperinflammation that results from an immunogenic trigger in the context of inadequate negative regulatory mechanisms (1). The most severe forms of such cytokine storm disorders are genetically defined syndromes caused by mutations in the granule exocytosis pathway, collectively known as familial hemophagocytic lymphohistiocytosis (FHL) (2). Among variants of FHL, mutations in more critical genes lead to a greater degree of impairment in CD8^+^ T cell and NK cell cytotoxicity and correlate with worsened disease; the most severe (and most common) form of FHL (type 2, FHL2) results from mutations in the gene encoding perforin (3, 4).

Analogous to FHL2 patients, perforin-deficient (*Prf1*^−/−^) mice develop lethal inflammation after viral infection; in this murine model of FHL2, uninhibited replication of lymphocytic choriomeningitis virus (LCMV) induces pathologic accumulation of LCMV-specific CD8^+^ T cells and interferon-γ (IFNγ) (5–7). We have previously shown that antigen-independent danger signals mediated through ST2, the interleukin-33 (IL-33) receptor, are necessary to amplify inflammation to fatal levels (8). Antibody blockade of ST2 dampens T cell-mediated production of IFNγ; however, the mechanism by which abrogation of ST2 signaling alters T cell responses, remains unclear. In particular, chronic defects in CD8^+^ T cell function have been implicated as a protective negative regulatory mechanism in the related murine model of FHL type 4 (FHL4) (9). In these syntaxin-11-deficient (*Stx11*^−/−^) mice, disease develops transiently following LCMV infection but gradually becomes quiescent as the CD8^+^ T cell pool undergoes a functional decline known as exhaustion. Whether a similar mechanism occurs in other murine models of FHL has not been shown.

CD8^+^ T cell exhaustion is a progressive, step-wise loss of effector functions that occurs in the context of persistently high levels of antigen. Diminished interleukin-2 (IL-2) production, followed by sequential loss of tumor necrosis factor-α (TNFα), IFNγ, and cytotoxicity, coincides with upregulation of inhibitory markers such as PD-1 (10–12). Exhausted CD8^+^ T cells gradually lose proliferative capacity and become increasingly susceptible to apoptosis, eventually undergoing deletion from the host altogether (13). While exhaustion poses an obstacle to the treatment of chronic infections and malignancy, it may also serve a protective role in limiting immunopathology (14, 15). In the case of murine FHL4, CD8^+^ T cell exhaustion has been implicated in prolonged survival (9); conversely, the CD8^+^ T cells in murine FHL2 fail to undergo exhaustion, leading to overwhelming inflammation and mortality.

In this study, we hypothesized that the long-term survival benefit of ST2 blockade in murine FHL2 is mediated through CD8^+^ T cell exhaustion, similar to unmanipulated murine FHL4. We found that in the setting of ST2 blockade, LCMV-specific CD8^+^ T cells in *Prf1*^−/−^ mice develop the phenotypic and functional hallmarks of exhaustion. However, reversal of exhaustion in the absence of ST2 signaling had no effect on mortality in murine FHL2, suggesting that CD8^+^ T cell exhaustion is a consequence, rather than a cause, of their non-fatal disease progression. Withdrawal of ST2 blockade failed to alter long-term outcomes, leading us to investigate the acute effects of ST2 blockade in further detail. We found that disruption of IL-33 signaling via ST2 blockade blunted the quality of the effector T cell response by intrinsically decreasing per-cell production of IFNγ and restricting CD8^+^ T cell proliferation. Our work therefore suggests that while ST2 blockade indirectly enables the development of CD8^+^ T cell exhaustion, its greatest therapeutic potential is in limiting T cell inflammation during the acute phase of disease.

## Materials and methods

### Mice

C57BL/6 (WT), perforin-deficient (C57BL/6-Prf1^tm1Sdz^/J, referred to as *Prf1*^−/−^), B6.SJL, and Rag1^−/−^ mice were purchased from The Jackson Laboratory. ST2-deficient *Il1rl1*^−/−^ mice, originally derived from Andrew McKenzie (University of Cambridge) (16) and back-crossed to C57BL/6, were kindly provided by Peter Nigrovic (Harvard University). All mice were bred and housed in an Association for Assessment and Accreditation of Laboratory Animal Care–certified animal facility, and all experiments were performed with approval of the Children's Hospital of Philadelphia Institutional Animal Care and Use Committee.

### Viral infection

Armstrong and Clone 13 strains of LCMV were kindly provided by E. John Wherry (University of Pennsylvania). To induce FHL, mice aged 7–9 weeks were infected i.p. with 2 × 10^5^ PFU LCMV-Armstrong and were euthanized upon development of significant morbidity or weight loss. WT and *Il1rl1*^−/−^ mice were infected with 4 × 10^6^ PFU LCMV-Clone 13 by retro-orbital i.v. injection. Viral titers were measured by plaque assays on Vero cells as previously described (17). Unless otherwise specified, LCMV refers to LCMV-Armstrong.

### *In vivo* treatments

Rat anti-mouse ST2-blocking antibody with muIgG1 Fc domain (α-ST2 antibody) and mouse IgG1 isotype control antibody were provided by Amgen and have been previously described (18). For ST2 blockade in *Prf1*^−/−^ mice, LCMV-infected mice were injected i.p. with 150 μg α-ST2 antibody or 150 μg Control antibody every other day, beginning on day 3 p.i, as previously described (8). Withdrawal from ST2 blockade was achieved by switching mice to Control antibody treatment after 15 days post-infection. α-PD-L1 antibody (clone 10F.9G2) and rat IgG2b isotype control antibody were purchased from BioXCell (10). For PD-L1 blockade, LCMV-infected mice were injected i.p. with 200 μg α-PD-L1 antibody or 200 μg Control antibody every 3 days, beginning on day 15 p.i.

### Bone marrow (BM) chimeras

Irradiated CD45.2^+^* Rag1*^−/−^ hosts received BM from CD45.1^+^* Prf1*^−/−^ donors, CD45.2^+^* Prf1*^−/−^*Il1rl1*^−/−^ donors, or a 1:1 mixture of both. T cell reconstitution was confirmed by cheek bleed 6 weeks post-transfer and FHL was induced 8 weeks post-transfer. A repeat experiment in irradiated *Prf1*^−/−^ hosts demonstrated similar findings as in *Rag*^−/−^ hosts (data not shown).

### Flow cytometry

Splenocytes were stained with LIVE/DEAD fixable viability dye (Thermo Fisher Scientific), Fc blocked (clone 2.4G2, produced in house), and stained with antibodies to CD4, CD8α, CD19, CD44, CD45.1, CD45.2, CD62L, CD90.2, NK1.1, PD-1, 2B4, TCRβ, IFNγ, IL-2, Ki-67, T-bet, Eomes, and/or TNFα (BD Biosciences, eBioscience, BioLegend, and Miltenyi Biotec). H-2D^b^GP_33−41_ MHC-peptide complexes were provided as fluorophore-conjugated tetramers by E. John Wherry. Cells were fixed and permeabilized using Cytofix/Cytoperm (BD Bioscience) or the Foxp3/Transcription Factor Fixation/Permeabilization kit (eBioscience. Data were acquired using a Miltenyi Biotec MACSQuant flow cytometer and analyzed using FlowJo software versions 9.8, 9.9, or X.07 (FlowJo, LLC).

### *In vitro* assays

Serum IFNγ was measured using OptEIA enzyme-linked immunosorbent assay (BD Biosciences). LCMV peptide restimulation assays were performed as previously described (8). For degranulation assays, PE-conjugated CD107a antibody and monensin were included in culture medium for the duration of the stimulation (19). Initiation of apoptosis was measured by incubation with Vybrant FAM-DEVD-FMK caspase-3 and −7 reagent, referred to as FLICA (FLuorescent Inhibitor of CAspases), according to manufacturer instructions (Thermo Fisher Scientific).

### Statistical analysis

Weight loss data were analyzed by linear mixed-effects models as previously described (8). All other data were analyzed in GraphPad Prism 5 using statistical tests indicated in figure legends. Unless otherwise specified, *P*-values are represented in figures by number of symbols (e.g., ^*^P < 0.05, ^**^P < 0.01, ^***^P < 0.001).

### Data sharing

The raw data supporting the conclusions in this manuscript will be made available by the authors, without undue reservation, to any qualified researcher.

## Results

### *Prf1^−/−^* LCMV-specific CD8^+^ T cells become exhausted in the setting of ST2 blockade

**Figure 1:**
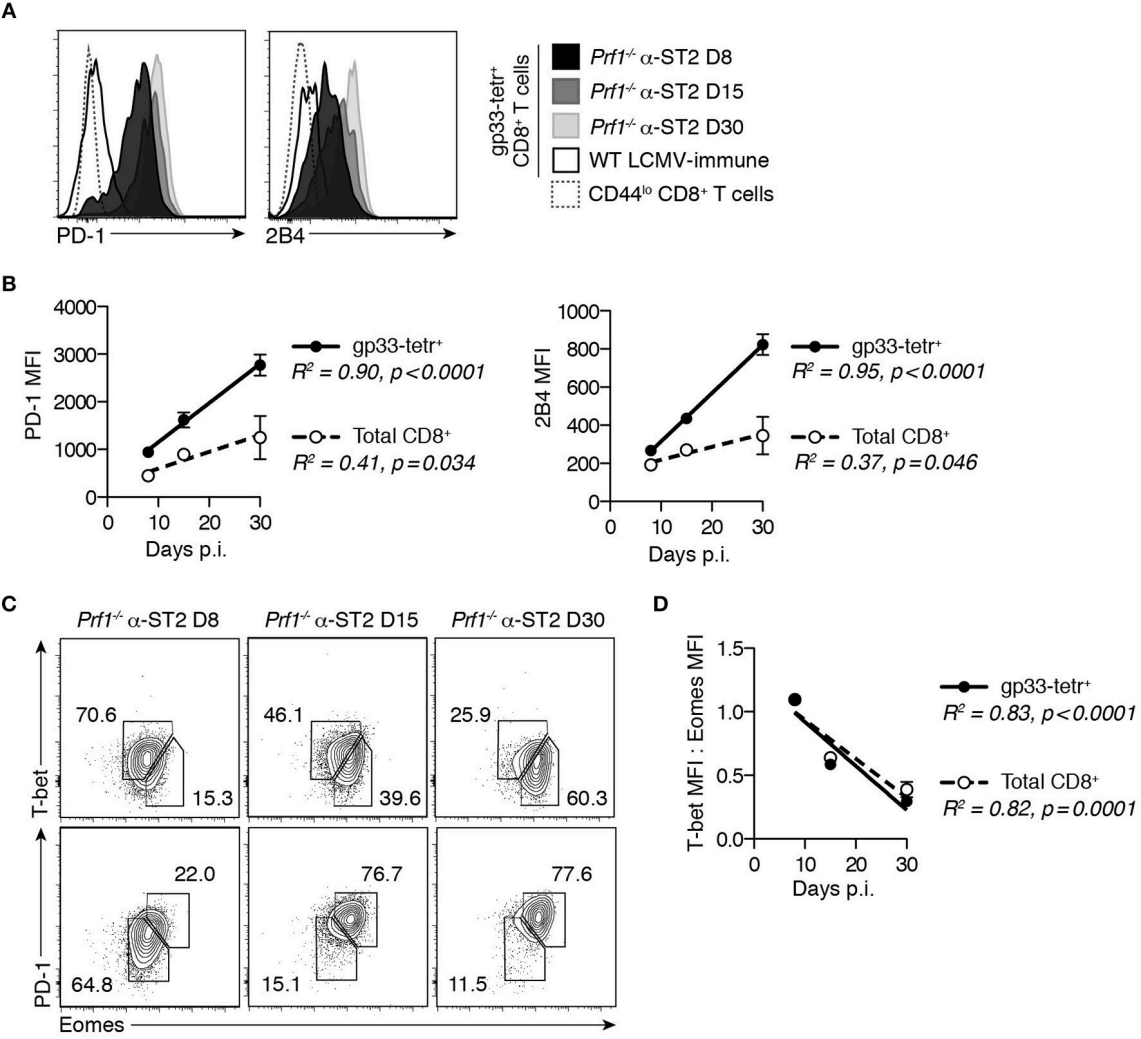
*Prf1*^−/−^ gp33-specific CD8^+^ T cells develop phenotypic hallmarks of exhaustion in the setting of ST2 blockade. *Prf1*^−/−^ mice were infected with LCMV and treated every other day with α-ST2 antibody beginning on day 3 p.i. Splenocytes were assessed on days 8, 15, or 30 p.i. WT mice infected with LCMV and analyzed 30 days p.i. provided an LCMV-immune control. *N* = 3–4 mice/group. **(A)** Representative histograms gated on gp33-tetramer^+^ CD8^+^ T cells, showing expression of inhibitory markers. **(B)** MFI of PD-1 and 2B4 in gp33-tetramer^+^ (filled symbols) and total (open symbols) CD8^+^ T cells over time. Symbols represent mean ± SEM of 3-4 mice. Analyzed by linear regression. **(C)** Representative flow plots gated on gp33-tetramer^+^ CD8^+^ T cells, showing expression of T-bet, Eomes, and PD-1. Numbers indicate the frequency of cells within the adjacent gate. **(D)** Ratio of T-bet MFI to Eomes MFI in gp33-tetramer^+^ (filled symbols) and total (open symbols) CD8^+^ T cells over time. Symbols represent mean ± SEM of 3–4 mice. Analyzed by linear regression.

Given the association of CD8^+^ T cell exhaustion with long-term survival in murine FHL4, we first determined whether the pro-survival effect of ST2 blockade similarly enables development of CD8^+^ T cell exhaustion in murine FHL2. The lethality of the FHL2 model precludes late-stage analysis of *Prf1*^−/−^ CD8^+^ T cell responses without introducing survival bias. We therefore conducted a longitudinal analysis of LCMV-specific CD8^+^ T cells from *Prf1*^−/−^ mice treated with ST2-blocking antibody (α-ST2) to investigate the evolution of the T cell response in the setting of ST2 blockade. α-ST2-treated *Prf1*^−/−^ mice demonstrated consistently elevated expression of the inhibitory markers PD-1 and 2B4 on gp33-specific CD8^+^ T cells following LCMV infection (Figure 1A). Median fluorescence intensity (MFI) of these markers increased gradually from days 8 to 30 post-infection (p.i.), suggesting progressive impairment (Figures 1A,B). Upregulation of inhibitory markers occurred globally within the CD8^+^ T cell pool but was most pronounced among LCMV-specific CD8^+^ T cells (Figure 1B). We next assessed differential expression of the key transcription factors T-bet and Eomesodermin (Eomes), which identifies two subsets of exhausted cells: a progenitor T-bet^hi^ Eomes^lo^ PD-1^int^ population and its terminally exhausted Eomes^hi^ T-bet^lo^ PD-1^hi^ progeny (20). In ST2-blocked *Prf1*^−/−^ mice, the majority of gp33-specific CD8^+^ T cells were T-bet^hi^ at day 8 p.i. but converted to Eomes^hi^ PD-1^hi^ by day 30 p.i. (Figure 1C), a transition evident by the gradual decrease in the ratio of T-bet:Eomes MFI among both LCMV-specific and total CD8^+^ T cells (Figure 1D). This evolution in phenotype and transcription factor expression among *Prf1*^−/−^ CD8^+^ T cells in the setting of ST2 blockade is consistent with the development of exhaustion.

**Figure 2:**
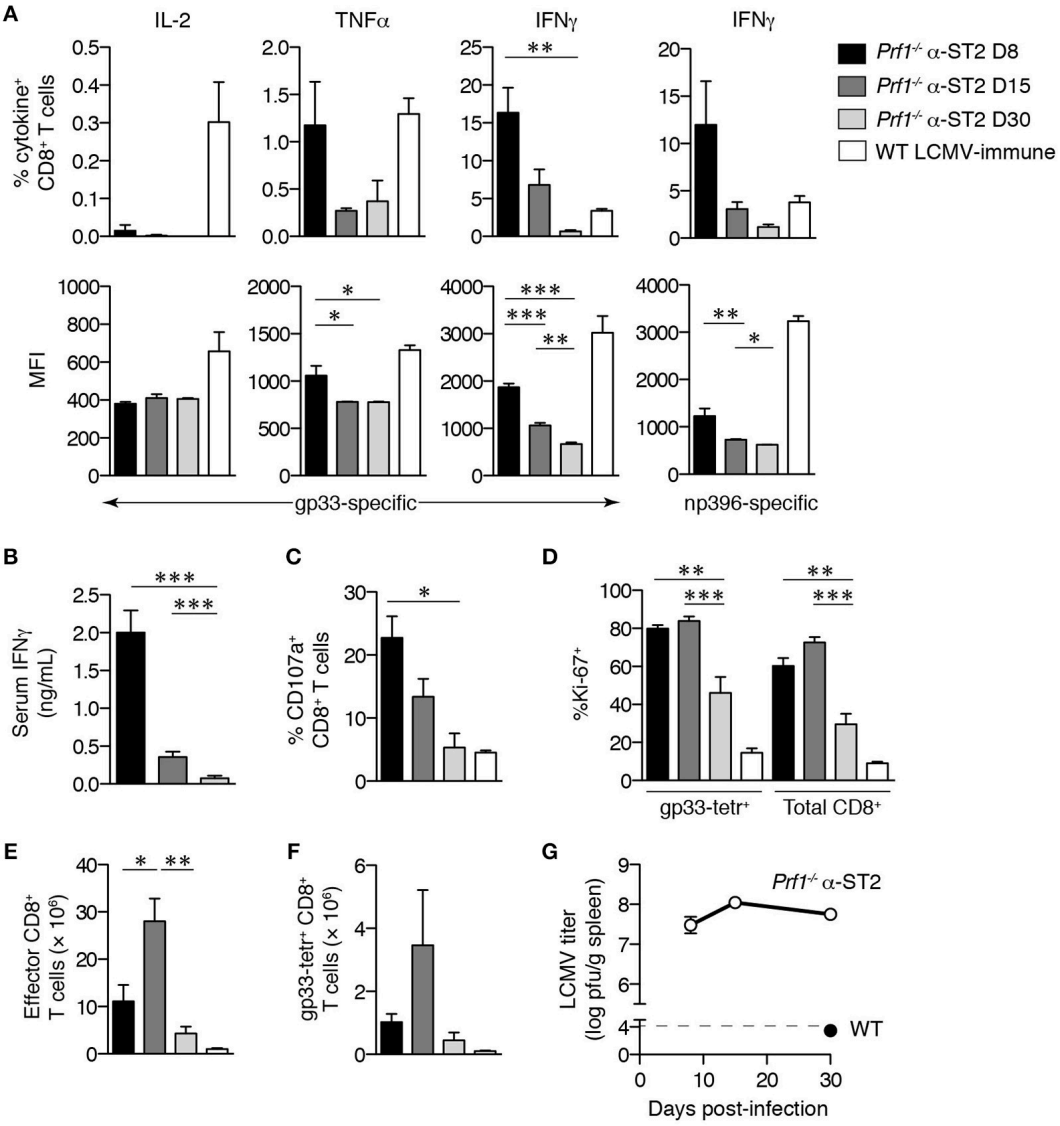
Gp33-specific CD8^+^ T cells in ST2-blocked *Prf1*^−/−^ mice become functionally exhausted in the context of persistent viremia. *Prf1*^−/−^ mice were infected with LCMV and treated every other day with α-ST2 antibody beginning on day 3 p.i. Splenocytes were assessed on days 8, 15, or 30 p.i. WT mice infected with LCMV and analyzed 30 days p.i. provided an LCMV-immune control. Bars and symbols represent mean ± SEM of 3-4 mice. *Prf1*^−/−^ α-ST2 groups were analyzed by one-way ANOVA; significance of Tukey's post-test comparing all groups is indicated. **(A)** Frequencies of CD8^+^ T cells specifically producing cytokine in response to *in vitro* gp33 or np396 peptide stimulation (top row) and MFI of cytokine^+^ CD8^+^ T cells (bottom row). **(B)** Serum IFNγ level. **(C)** Frequencies of CD8^+^ T cells specifically externalizing CD107a in response to *in vitro* gp33 peptide stimulation. **(D)** Frequencies of gp33-tetramer^+^ and total CD8^+^ T cells expressing Ki-67. **(E)** Numbers of splenic effector (CD44^hi^CD62L^lo^) CD8^+^ T cells. **(F)** Numbers of gp33-specific CD8^+^ T cells. **(G)** Splenic LCMV titer. Dotted line indicates lower limit of detection of plaque assay.

To determine whether these changes correlate with true functional exhaustion, we assessed cytokine production, cytotoxicity, and proliferation of *Prf1*^−/−^ CD8^+^ T cells from ST2-blocked mice. At day 8 p.i., gp33-specific CD8^+^ T cells displayed negligible IL-2 production and low TNFα production, which decreased further by day 15 p.i. (Figure 2A). IFNγ production capacity was initially robust, but both the frequency of responding gp33-specific cells and the potency of their response decreased progressively over time (Figure 2A), coinciding with a decline in serum IFNγ (Figure 2B). This phenomenon was not epitope-specific, as a similar decline in IFNγ production was also evident among np396-specific CD8^+^ T cells (Figure 2A, right column). Of note, the median per-CD8^+^ T cell expression of cytokines at 30 days p.i. was consistently lower in α-ST2-treated *Prf1*^−/−^ mice than LCMV-immune WT mice (Figure 2A), suggesting failed development of functional memory CD8^+^ T cell responses. Although *Prf1*^−/−^ cells are incapable of mediating cytotoxicity, gp33-specific CD8^+^ T cells from ST2-blocked *Prf1*^−/−^ mice demonstrated reduced degranulation, a proxy for impaired functional capacity (Figure 2C). The frequency of Ki-67^+^ proliferating cells among gp33-specific and total CD8^+^ T cells also decreased after day 15 p.i. (Figure 2D), coinciding with a precipitous decline in the numbers of both bulk effector and gp33-specific CD8^+^ T cells (Figures 2E,F). In contrast, total numbers of naïve and memory CD8^+^ T cells did not change appreciably over the course of infection, with the exception of a modest decrease in memory CD8^+^ T cells from day 15 to day 30 p.i. (*P* < 0.01, data not shown). This contraction of the LCMV-specific CD8^+^ T cell pool and global loss of effector function was not due to viral clearance, since ST2-blocked *Prf1*^−/−^ mice sustained high splenic LCMV titers throughout (Figure 2G), consistent with previous reports of the inability of *Prf1*^−/−^ mice to eliminate LCMV (7). These data collectively demonstrate the hierarchical loss of function that defines CD8^+^ T cell exhaustion and establish the ability of *Prf1*^−/−^ CD8^+^ T cells to undergo exhaustion in the setting of ST2 blockade.

### CD8^+^ T cell exhaustion is not a direct effect of ST2 blockade in LCMV-infected mice

**Figure 3:**
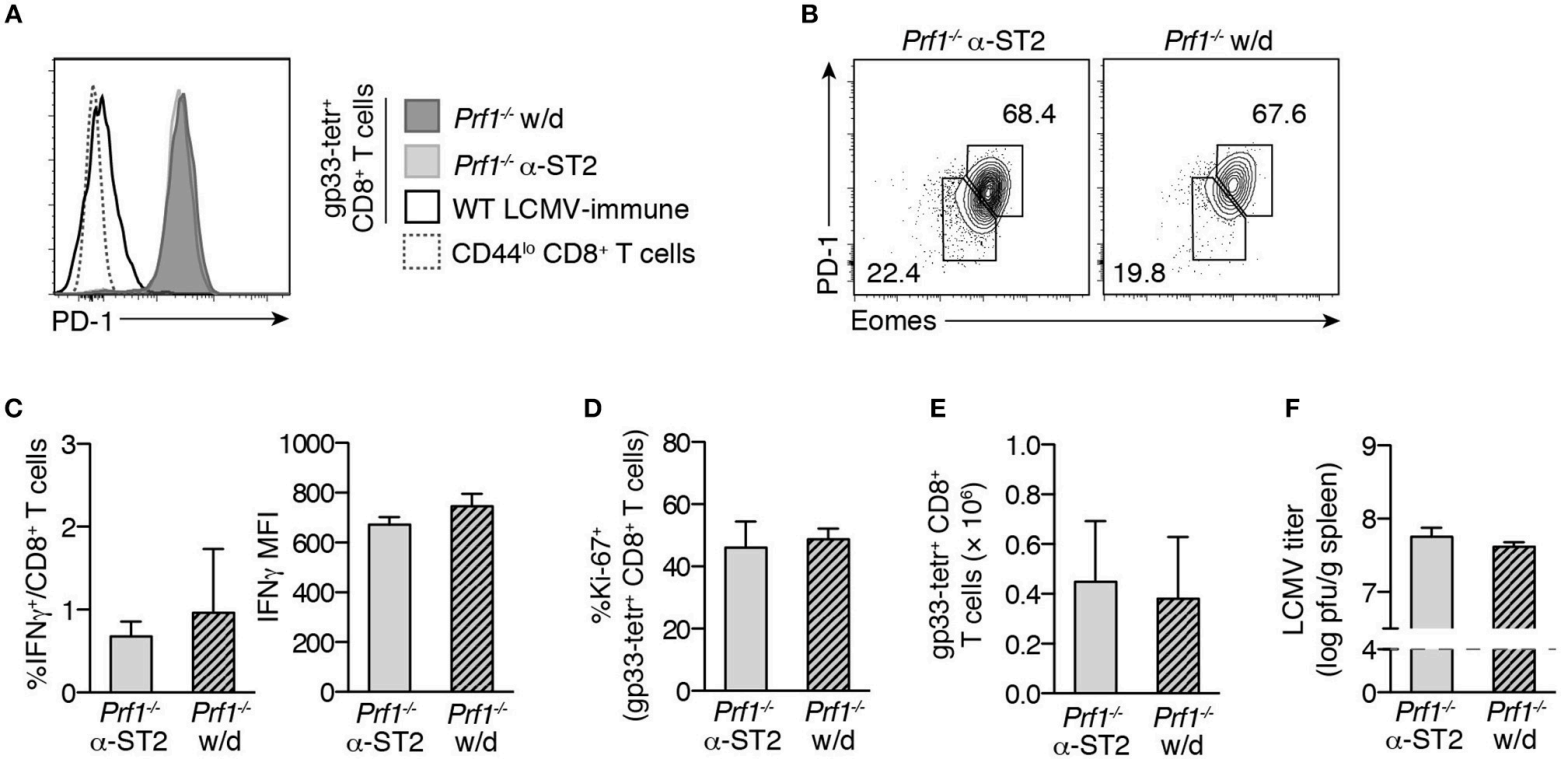
Withdrawal of ST2 blockade does not reverse CD8^+^ T cell exhaustion. *Prf1*^−/−^ mice were infected with LCMV and either treated with α-ST2 antibody continually (α-ST2) or were withdrawn (w/d) from α-ST2 treatment after day 15 p.i. and thereafter switched to isotype control antibody. Antibody treatments were administered every other day, beginning on day 3 p.i. Splenocytes were assessed on day 30 p.i. *N* = 3–4 mice/group. Bars represent mean ± SEM. Analyzed by Student's two-tailed *t*-test. **(A)** Representative histograms gated on gp33-tetramer^+^ CD8^+^ T cells, showing expression of PD-1. **(B)** Representative flow plots gated on gp33-tetramer^+^ CD8^+^ T cells, showing expression of Eomes and PD-1. Numbers indicate the frequency of cells within the adjacent gate. **(C)** Frequency and IFNγ MFI of CD8^+^ T cells specifically producing IFNγ in response to *in vitro* gp33 peptide stimulation. **(D)** Frequencies of gp33-tetramer^+^ CD8^+^ T cells expressing Ki-67. **(E)** Numbers of splenic gp33-specific CD8^+^ T cells. **(F)** Splenic LCMV titer. Dotted line indicates lower limit of detection of plaque assay.

We had previously shown that mice withdrawn from ST2 blockade after 2 weeks of infection were able to maintain similar survival to mice that remained on blockade for 30 days (8). However, these same mice, when withdrawn from ST2 blockade, did show a significant weight loss compared to mice maintained on blockade (8). If ST2 blockade directly promotes CD8^+^ T cell exhaustion in murine FHL2, then withdrawal of ST2 blockade might be expected to lessen the severity of exhaustion, consistent with increased immune activation that might account for this weight loss. However, gp33-specific CD8^+^ T cells of *Prf1*^−/−^ mice withdrawn from ST2 blockade had equally high expression of PD-1 and 2B4 as those of mice remaining on α-ST2 treatment (Figure 3A and data not shown). Similar frequencies of gp33-specific CD8^+^ T cells segregated with the Eomes^hi^ PD-1^hi^ subset of terminally exhausted cells in the presence or absence of continual α-ST2 treatment (Figure 3B). Moreover, withdrawal of ST2 blockade did not alter effector function, as both groups demonstrated equivalently low frequencies of IFNγ^+^ gp33-specific cells and low per-cell IFNγ production (Figure 3C). The frequency and expression level of TNFα and IL-2 were also unaffected, as was degranulation (data not shown) and frequencies of proliferating Ki-67^+^ cells (Figure 3D). Accordingly, similarly low numbers of total effector and gp33-specific CD8^+^ T cells were observed after withdrawal of α-ST2 treatment (Figure 3E and data not shown), despite equivalently high viral loads (Figure 3F). We acknowledge that there may be some remaining ST2 blocking antibody in circulation even after withdrawal; however, the fact that withdrawn mice begin to lose weight after withdrawal (8) suggests that the pharmacodynamic effect of the drug does begin to wear off soon after withdrawal. Together, these new findings suggest that full CD8^+^ T cell exhaustion develops even in the absence of continual ST2 blockade, consistent with the notion that exhaustion develops as a secondary effect of initial disrupted ST2 signaling.

To determine whether ST2 impacts the development of CD8^+^ T cell exhaustion in general, we used a classic exhaustion model. The Armstrong and Clone 13 strains of LCMV differ by only two amino acids but produce divergent immune responses in wild type C57BL/6 mice. While WT mice are able to survive infection with either strain, Armstrong (Arm) virus is ably cleared within 8 days and induces robust memory CD8^+^ T cell responses, whereas infection with Clone 13 (Cl-13) produces a chronic viremic state and leads to CD8^+^ T cell exhaustion beginning 1–2 weeks p.i. (17, 21, 22). To investigate the relationship between ST2 signaling and CD8^+^ T cell exhaustion, we infected WT and ST2-deficient *Il1rl1*^−/−^ mice with Cl-13 and compared their CD8^+^ T cell responses to the functional effector CD8^+^ T cell response of Arm-infected WT mice. Numbers of effector and gp33-specific CD8^+^ T cells, inhibitory receptor expression, frequency of Ki-67^+^ cells, Eomes expression, cytokine production, and overall clinical outcome as measured by chronic weight loss, were similar in Cl-13-infected *Il1rl1*^−/−^ mice compared to WT mice (Figure S1). Importantly, CD8^+^ T cell responses in both groups of Cl-13-infected mice differed markedly from Arm-infected mice (Figure S1). Overall, the LCMV-specific CD8^+^ T cell response to Cl-13 infection was comparably exhausted in *Il1rl1*^−/−^ and WT mice, demonstrating that disruption of ST2 signaling does not directly impact the development of CD8^+^ T cell exhaustion. Interestingly, *Il1rl1*^−/−^ mice were significantly protected from early weight loss after Cl-13 infection (Figure S1H), suggesting that ST2 may be more important in the acute, rather than chronic, response to viral infection. This is consistent with our data in *Prf1*^−/−^ mice showing that disruption of ST2 signaling is only required early in the course of disease for the eventual development of CD8^+^ T cell exhaustion. In summary, these data suggest that absence of ST2 signaling promotes exhaustion in an indirect manner.

### CD8^+^ T cell exhaustion in LCMV-infected *prf1^−/−^* mice provides little clinical benefit

**Figure 4:**
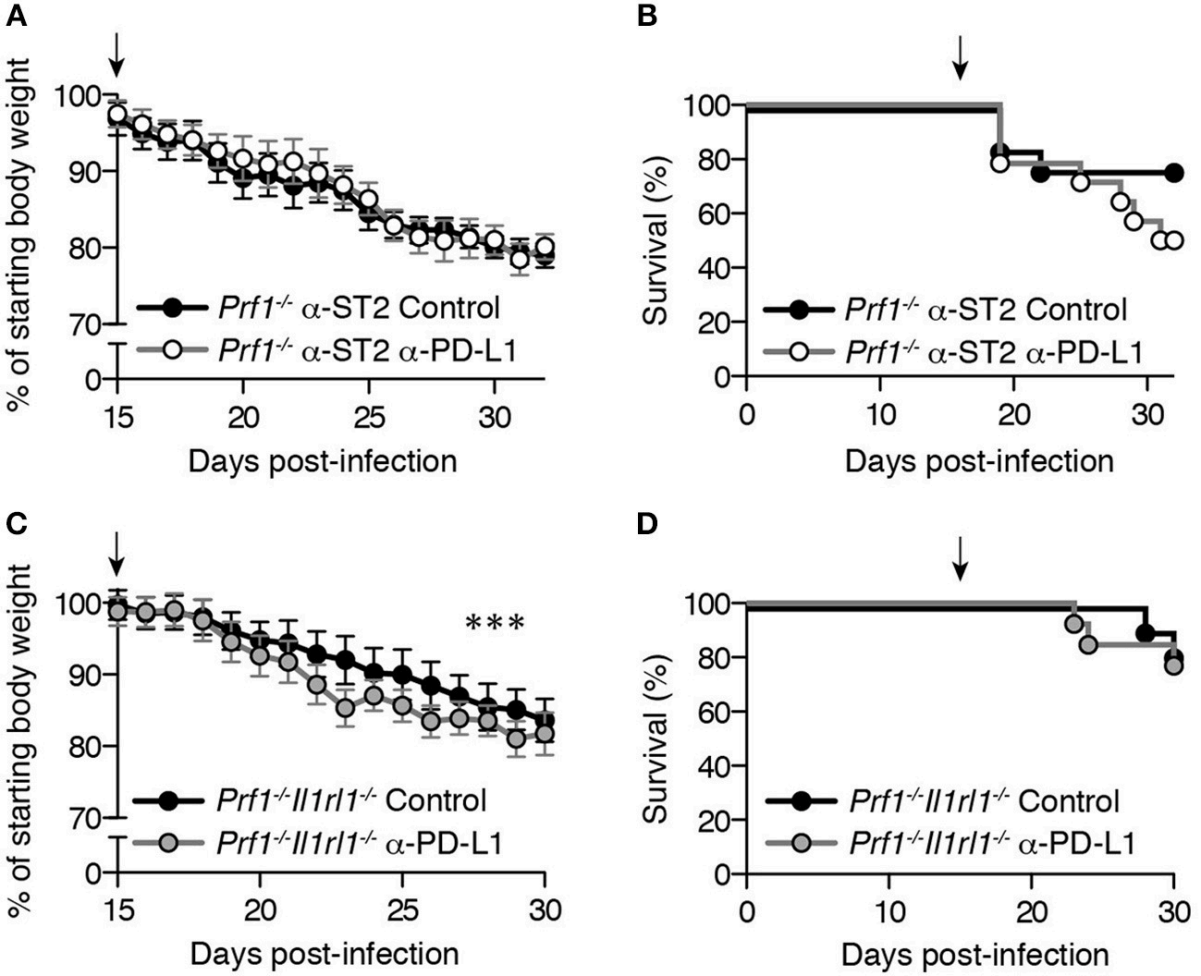
CD8^+^ T cell exhaustion in LCMV-infected *Prf1*^−/−^ mice provides little clinical benefit. Mice were infected with LCMV and given 200 μg of either α-PD-L1 or isotype control antibodies every 3 days, beginning on day 15 p.i. (indicated by an arrow). Day 15 was selected as the timepoint at which initial features of exhaustion are clearly evident(21) and PD-1 is highly upregulated on LCMV-specific *Prf1*^−/−^ CD8^+^ T cells (Figure 1A). Starting body weight was determined on day 0, and average weight did not differ significantly between the two groups from days 0 to 15 p.i. (not shown). Weight data are plotted as mean ± SEM and were analyzed by linear mixed-effects model. Survival data were analyzed by log-rank (Mantel-Cox) test. **(A)** Body weight and **(B)** survival of *Prf1*^−/−^ mice acutely treated with α-ST2 antibody every other day from days 3–15 p.i. prior to administration of α-PD-L1 or isotype control antibodies. *N* = 13–14 mice/group, pooled from 2 independent experiments. **(C)** Body weight and **(D)** survival of *Prf1*^−/−^*Il1rl1*^−/−^ mice receiving either α-PD-L1 or isotype control antibodies. *N* = 11–13 mice/group, pooled from 2 independent experiments.

Exhaustion plays an important role in resolving FHL in *Stx11*^−/−^ mice (9). To determine whether CD8^+^ T cell exhaustion promotes similar late-stage clinical improvement in murine FHL2, we pharmacologically reversed CD8^+^ T cell exhaustion using α-PD-L1 antibody, which blocks the interaction of PD-1 with one of its ligands and transiently enhances the effector function of exhausted CD8^+^ T cells (Figure S2) (10, 11). There was no difference in late-stage weight loss (Figure 4A) or survival (Figure 4B) between ST2-blocked *Prf1*^−/−^ mice receiving PD-L1 blockade compared to isotype control antibody, suggesting that exhaustion does not protect against chronic morbidity or mortality in murine FHL2.

To eliminate the possibility that incomplete ST2 blockade may have produced these results, we assessed the role of exhaustion in *Prf1*^−/−^ mice lacking ST2 (*Prf1*^−/−^*Il1rl1*^−/−^ mice, discussed below). *Prf1*^−/−^*Il1rl1*^−/−^ mice receiving PD-L1 blockade demonstrated transiently greater weight loss (Figure 4C), although the magnitude of this difference was small (mean weight difference−2.6%). More importantly, PD-L1 blockade did not enhance mortality among *Prf1*^−/−^*Il1rl1*^−/−^ mice (Figure 4D), similar to ST2-blocked mice. Comparable results were obtained in *Prf1*^−/−^*Il1rl1*^−/−^ mice using a protocol identical to that of Kögl et al. (9), with a small but statistically significant decrease in weight after double PD-L1/LAG-3 blockade (mean weight difference −2.4%, *P* < 0.0001) but no difference in mortality (data not shown). Thus, in contrast to findings in murine FHL4, CD8^+^ T cell exhaustion does not provide a significant clinical benefit in ST2-blocked *Prf1*^−/−^ mice. Exhaustion is therefore more likely a consequence, rather than a cause, of prolonging survival in *Prf1*^−/−^ mice via ST2 blockade.

### Inhibition of IL-33 signaling leads to early global defects in *prf1^−/−^* CD8^+^ T cell effector function

**Figure 5:**
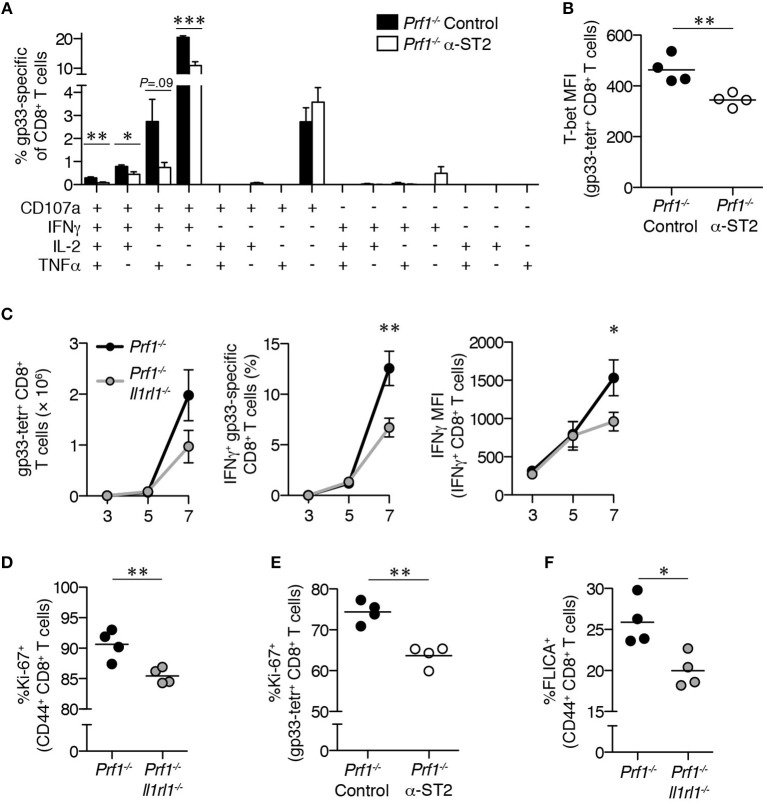
*Prf1*^−/−^ CD8^+^ T cells demonstrate early global defects in effector function in the absence of ST2 signaling. LCMV-infected *Prf1*^−/−^ mice were treated with either isotype control antibody (*Prf1*^−/−^ Control, shown in black bars/symbols) or ST2-blocking antibody (*Prf1*^−/−^ α-ST2, shown in white bars/symbols) and splenocytes were analyzed 8 days p.i. *N* = 4 mice/group. Analyzed by Student's two-tailed *t*-test. **(A)** Frequency of CD8^+^ T cells externalizing CD107a and/or producing IFNγ, IL-2, and/or TNFα in response to gp33 peptide (above background). **(B)** T-bet MFI in gp33 tetramer^+^ CD8^+^ T cells. **(C)** Total numbers of splenic gp33-tetramer^+^ CD8^+^ T cells (left panel), frequencies of IFNγ^+^ gp33-specific CD8^+^ T cells (middle panel), and median IFNγ fluorescence intensity of IFNγ^+^ CD8^+^ T cells (right panel) from LCMV-infected *Prf1*^−/−^ and *Prf1*^−/−^*Il1rl1*^−/−^ mice analyzed over time. Symbols represent mean ± SEM of *n* = 3–8 mice/timepoint/genotype. Data are pooled from 2 independent experiments. Day 7 timepoint analyzed by Student's two-tailed *t*-test. **(D)** Frequencies of Ki-67^+^ cells among *Prf1*^−/−^ or *Prf1*^−/−^*Il1rl1*^−/−^ CD44^+^CD8^+^ T cells. *N* = 4 mice/genotype. Analyzed at day 7 p.i. by Student's two-tailed *t*-test. **(E)** Frequencies of Ki-67^+^ gp33 tetramer^+^ CD8^+^ T cells from LCMV-infected *Prf1*^−/−^ mice treated with either isotype control antibody (*Prf1*^−/−^ Control) or ST2-blocking antibody (*Prf1*^−/−^ α-ST2). *N* = 4 mice/genotype. Analyzed at day 8 p.i. by Student's two-tailed *t*-test. **(F)** Frequencies of *Prf1*^−/−^ or *Prf1*^−/−^*Il1rl1*^−/−^ CD44^+^CD8^+^ T cells staining positively with fluorescent inhibitor of caspases 3 and 7 (FLICA). *N* = 4 mice/genotype. Analyzed at day 7 p.i. by Student's two-tailed *t*-test.

Our data suggest that loss of ST2 signaling has greater impact early in the course of disease than at later timepoints, turning our attention to acute effects of ST2 blockade on *Prf1*^−/−^ CD8^+^ T cell responses. At 8 day p.i., lower frequencies of polyfunctional gp33-specific *Prf1*^−/−^ CD8^+^ T cells were induced in mice receiving α-ST2 antibody (Figure 5A), and these cells demonstrated decreased expression of T-bet, suggesting a more global defect in effector differentiation and function in the absence of ST2 signaling (Figure 5B).

To further dissect the role of ST2 at early timepoints, we generated mice deficient in both perforin and ST2 (*Prf1*^−/−^*Il1rl1*^−/−^ mice). These mice were viable, displayed no overt alterations in appearance or behavior, and showed normal baseline frequencies and numbers of major immune cell populations (Figure S3 and data not shown). When infected with LCMV to induce FHL, *Prf1*^−/−^*Il1rl1*^−/−^ mice fully recapitulated the effects of α-ST2 antibody blockade in *Prf1*^−/−^ mice, including early defects in CD8^+^ IFNγ production and progression to exhaustion (Figure S4). It is worth noting that since *Prf1*^−/−^*Il1rl1*^−/−^ mice lacking both the soluble and membrane-bound forms of ST2 phenocopy α-ST2 treatment, the protective effects of ST2 blockade must be attributable to direct inhibition of IL-33 signaling, rather than potentiation of IL-33 signaling via inhibition of soluble ST2.

Timecourse analysis of *Prf1*^−/−^ and *Prf1*^−/−^*Il1rl1*^−/−^ mice revealed identical numbers of gp33-specific CD8^+^ T cells, frequencies of these cells producing IFNγ, and their per-cell IFNγ expression through 5 days p.i. (Figure 5C). Disparities emerged thereafter, with significantly decreased IFNγ production capacity and a trend toward fewer LCMV-specific cells in the absence of ST2 signaling by day 7 p.i. (*P* = 0.14; Figure 5C). These differences coincide with the timing of ST2 upregulation on CD8^+^ T cells in LCMV-infected WT mice (23). This time period is also marked by extensive proliferation of effector CD8^+^ T cells in response to LCMV (24), and we found that the frequency of antigen-experienced CD8^+^ T cells expressing Ki-67 was decreased in *Prf1*^−/−^*Il1rl1*^−/−^ mice compared to *Prf1*^−/−^ mice (Figure 5D). Reduced frequencies of Ki-67^+^ cells were also observed among *Prf1*^−/−^ gp33-specific CD8^+^ T cells during α-ST2 treatment (Figure 5E). These proliferative defects were unlikely to result from enhanced cell death, since the frequency of CD8^+^ T cells induced to undergo apoptosis was decreased in *Prf1*^−/−^*Il1rl1*^−/−^ mice relative to *Prf1*^−/−^ mice (Figure 5F). These data demonstrate that loss of IL-33 signaling impairs both cytokine production and the proliferative burst among CD8^+^ T cells in murine FHL2.

### LCMV-specific *prf1^−/−^* T cells intrinsically require ST2 for enhanced expansion and IFNγ production

**Figure 6:**
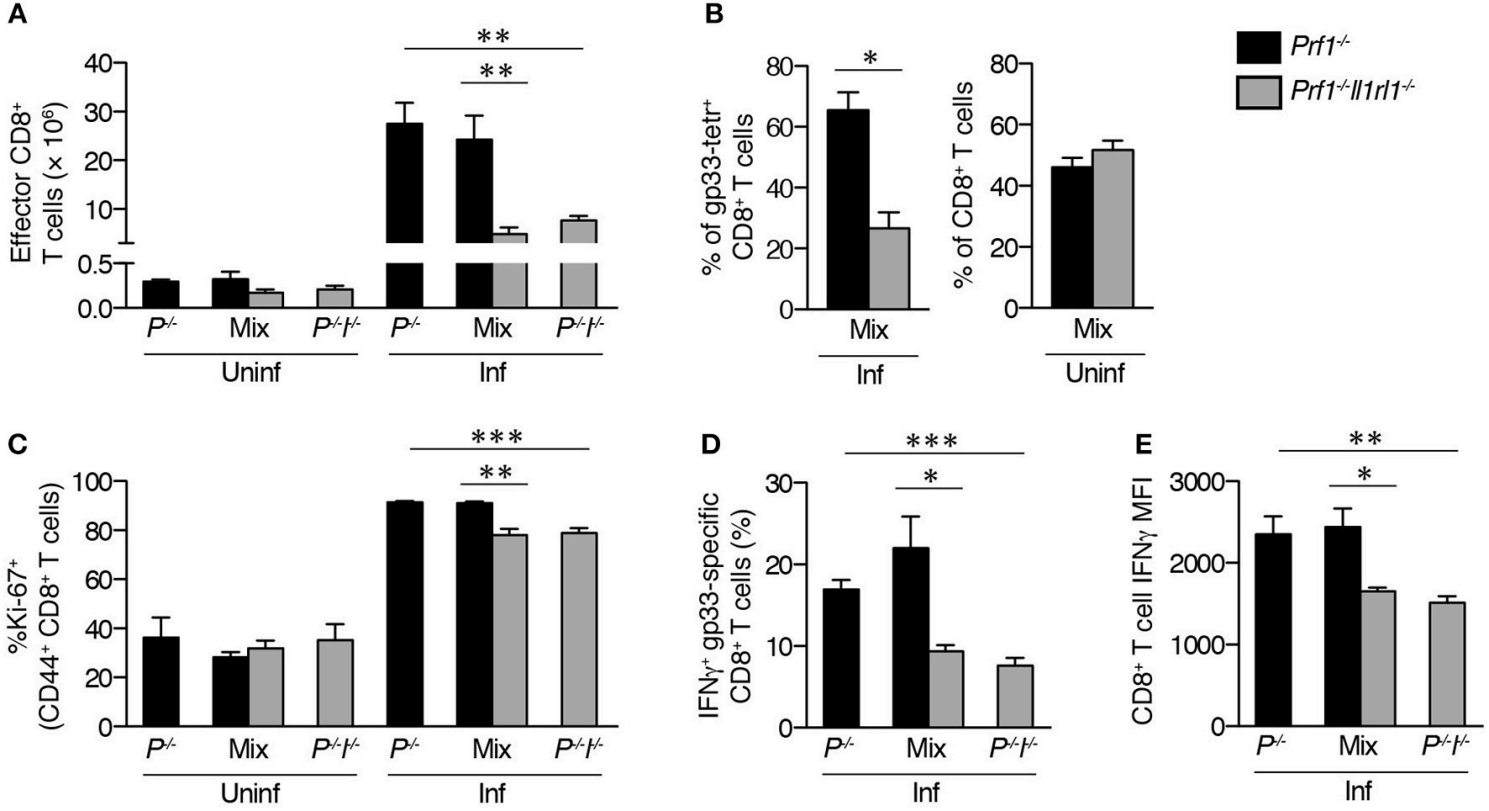
LCMV-specific *Prf1*^−/−^ T cells intrinsically require ST2 for enhanced expansion and IFNγ production. BM chimeras reconstituted with cells from *Prf1*^−/−^ donors (*Prf1*^−/−^), *Prf1*^−/−^*Il1rl1*^−/−^ donors (*P*^−/−^*I*^−/−^), or a 1:1 mixture (Mix) were either left uninfected (Uninf) or were infected with LCMV (Inf) and analyzed 8 days p.i. *N* = 4–5 mice/group. Single-donor chimeras were analyzed by unpaired Student's two-tailed *t*-test (*P*^−/−^* vs. P*^−/−^*I*^−/−^); *Prf1*^−/−^ and *Prf1*^−/−^*Il1rl1*^−/−^ splenocytes within mixed chimeras were analyzed by paired Student's two-tailed *t*-test. **(A)** Numbers of CD44^+^CD62L^−^ effector CD8^+^ T cells. **(B)** Frequencies of *Prf1*^−/−^ or *Prf1*^−/−^*Il1rl1*^−/−^ cells among gp33 tetramer^+^ CD8^+^ T cells in infected mixed chimeras (left), or among total CD8^+^ T cells in uninfected mixed chimeras (right). **(C)** Frequencies of Ki-67^+^ cells among CD44^+^ CD8^+^ T cells derived from *Prf1*^−/−^ or *Prf1*^−/−^*Il1rl1*^−/−^ donors. **(D)** Frequencies of IFNγ^+^ LCMV-specific CD8^+^ T cells. **(E)** Median IFNγ fluorescence intensity of CD8^+^ T cells producing IFNγ in response to restimulation with LCMV gp33 peptide.

Finally, we determined whether proliferative and cytokine production defects in the absence of IL-33 signaling were CD8^+^ T cell-intrinsic or –extrinsic. WT CD8^+^ T cells upregulate ST2 and intrinsically require expression of this receptor for optimal expansion in response to LCMV, but it is unknown whether IL-33 exerts the same effects on *Prf1*^−/−^ cells or is required for IFNγ production (25). Using mixed bone marrow (BM) chimeras, we found reduced numbers of effector CD8^+^ T cells derived from *Prf1*^−/−^*Il1rl1*^−/−^ BM compared to *Prf1*^−/−^ BM after induction of FHL (Figure 6A). The proportion of *Prf1*^−/−^*Il1rl1*^−/−^ cells within the gp33-specific subset was also markedly reduced relative to *Prf1*^−/−^ cells after infection, despite similar baseline ratios of *Prf1*^−/−^ to *Prf1*^−/−^*Il1rl1*^−/−^ CD8^+^ T cells in uninfected mixed BM chimeras (Figure 6B). This expansion defect corresponded to lower frequencies of *Prf1*^−/−^*Il1rl1*^−/−^ effector CD8^+^ T cells expressing Ki-67 than *Prf1*^−/−^ cells within the same infected hosts, suggesting an intrinsic proliferative defect (Figure 6C). Of note, there were no differences in Ki-67 expression or effector cell number in the absence of LCMV infection (Figures 6A,C). Thus, *Prf1*^−/−^ CD8^+^ T cells lacking ST2 demonstrate a cell-intrinsic defect in proliferation leading to decreased overall expansion.

As expected, decreased frequencies of IFNγ^+^ gp33-specific CD8^+^ T cells were observed in *Prf1*^−/−^*Il1rl1*^−/−^ single donor-derived BM chimeras compared to *Prf1*^−/−^ donor-derived chimeras (Figure 6D). Importantly, this disparity was recapitulated within mixed BM chimeras, indicating a CD8^+^ T cell-intrinsic reliance on ST2 (Figure 6D). Furthermore, median per-cell IFNγ expression was intrinsically reduced in IFNγ^+^* Prf1*^−/−^*Il1rl1*^−/−^ CD8^+^ T cells relative to their *Prf1*^−/−^ counterparts (Figure 6E). Together, these data show that inhibition of IL-33 signaling intrinsically dampens *Prf*^−/−^ CD8^+^ T cell effector function early in the course of disease and suggest that the greatest impact of ST2 blockade in murine FHL2 is in restricting both the quality and the quantity of the acute pathologic CD8^+^ T cell response.

## Discussion

In this study, we show that blockade of IL-33 signaling intrinsically attenuates proliferation and cytokine production of LCMV-specific CD8^+^ T cells in murine FHL2 early in the course of disease. This acute impairment in the quality of the effector CD8^+^ T cell response leads to reductions in the quantity of both disease-mediating effector CD8^+^ T cells and systemic levels of IFNγ, thereby dampening an otherwise lethal inflammatory response (8). Furthermore, ST2 blockade indirectly enables the development of CD8^+^ T cell exhaustion in murine FHL2 via its effects on prolongation of survival. However, unlike the FHL4 model, these chronic T cell functional defects offer little clinical benefit. Rather, the acute effects of ST2 blockade in limiting CD8^+^ T cell expansion and preventing accumulation of supraphysiologic levels of IFNγ confer greater impact in this disease model through mitigation of early morbidity and mortality.

Our findings reveal a cell-intrinsic ST2 signaling requirement of CD8^+^ T cells for effector expansion and IFNγ overproduction in FHL2 mice. This is consistent with previously published reports in LCMV-infected perforin-sufficient mice, showing that the accumulation and function of WT CD8^+^ T cells is regulated by ST2 in a cell-intrinsic manner (23). However, whereas Bonilla et al. implicated increased susceptibility to apoptosis in the failure of LCMV-specific ST2-deficient CD8^+^ T cells to accumulate in response to LCMV, we found decreased activation of caspases 3 and 7 in *Prf1*^−/−^*Il1rl1*^−/−^ CD44^+^CD8^+^ T cells relative to *Prf1*^−/−^ cells. Furthermore, we noted intrinsically reduced Ki-67 expression among LCMV-specific *Prf1*^−/−^*Il1rl1*^−/−^ CD8^+^ T cells, indicating that lower proliferative capacity, rather than enhanced apoptosis, likely accounted for the decreased number of *Prf1*^−/−^ effector CD8^+^ T cells in the absence of ST2 signaling. While these divergent results may be attributed to differences in types of CD8^+^ T cells analyzed, it is also possible that intrinsically dysregulated *Prf1*^−/−^ CD8^+^ T cells (25) react differently to the loss of ST2 signaling than do perforin-sufficient cells.

CD8^+^ T cells also intrinsically required ST2 for elevated per-cell production of IFNγ. Whether these functions are enhanced by proximal effects of ST2 signaling or represent distal manifestations of ST2-dependent alterations in effector T cell differentiation remains unclear. ST2 activates several downstream pathways, including NF-κB, which is necessary for Th1 effector differentiation and cytotoxic CD8^+^ T cell function (26, 27), as well as p38 MAPK, which is required by both CD8^+^ and CD4^+^ T cells for optimal IFNγ production (28). Further investigation of the intracellular pathways responsible for the stimulatory effects of IL-33 in murine FHL may offer insight into these questions.

This study establishes that development of exhaustion in FHL is not restricted to *Stx11*^−/−^ mice, since *Prf1*^−/−^ CD8^+^ T cells are also capable of undergoing exhaustion under certain conditions. Prior studies have suggested the possibility of CD8^+^ T cell exhaustion occurring in *Prf1*^−/−^ mice but have failed to definitively demonstrate this in the absence of survivor bias (29, 30). We show that in the context of ST2 blockade, *Prf1*^−/−^ CD8^+^ T cell uniformly undergo exhaustion. Strikingly, unlike *Stx11*^−/−^ mice, *Prf1*^−/−^ mice lacking ST2 show no increase in mortality after exhaustion reversal. This may indicate a major difference between murine FHL2 and FHL4, highlighting the pitfalls of extrapolating from one model of hemophagocytic lymphohistiocytosis to another. Rather than exhaustion enabling survival, as in murine FHL4, ST2 blockade in murine FHL2 likely represents the converse: prolonged survival gives CD8^+^ T cells time to undergo exhaustion. Critical care measures used to support human FHL patients in the initial stages of cytokine storm similarly extend survival, although whether CD8^+^ T cell exhaustion occurs in these patients is unknown, due to the rapid initiation of immunosuppressive therapy upon diagnosis (31, 32).

We therefore propose a conceptual model to explain the relationship between CD8^+^ T cell responsiveness and survival in murine FHL2, in which disease outcome is determined by the kinetics of inflammation in the context of the entire organism. *Prf1*^−/−^ mice succumb to LCMV infection not because of a failure of exhaustion, but because the amplitude of the acute CD8^+^ T cell response rapidly overwhelms the body's compensatory mechanisms, leading to early death from immunopathologic damage. However, disruption of ST2 signaling suppresses inflammation below the threshold of mortality, thereby prolonging CD8^+^ T cell exposure to virus. Faced with persistently high antigen levels and given sufficient time, CD8^+^ T cells inevitably undergo exhaustion (33, 34). Such a model could account for the fact that exhaustion has not previously been observed in the majority of *Prf1*^−/−^ mice. It provides a potential explanation for the development of CD8^+^ T cell exhaustion in milder disease phenotypes, such as syntaxin-11 deficiency (4, 9), although disease-specific differences cannot be ruled out. Furthermore, this model suggests that the primary therapeutic benefit of ST2 blockade derives not from an ability to induce exhaustion, but to limit early immunopathology. It is likely that other interventions capable of blunting inflammation and extending survival—whether neutralization of IFNγ, JAK1/2 signaling, LCMV, or some other factor (6, 35)—would have a similar effect as inhibition of IL-33 signaling.

In summary, we have shown that ST2 blockade dampens acute inflammation in murine FHL2 by intrinsically limiting IL-33-dependent effector CD8^+^ T cell function. Although ST2 blockade also promotes chronic alterations to the immune response—namely, CD8^+^ T cell exhaustion – these changes likely represent downstream side effects of restricting IL-33 signaling and are less important for disease outcome than the acute response. Early administration of ST2-blocking antibodies may therefore provide therapeutic benefit in FHL2 patients through diminution of systemic IFNγ by directly limiting its production by activated T cells. Our work thus suggests that pharmacologic inhibition of the IL-33/ST2 axis early in disease may offer a promising therapeutic strategy for the treatment of FHL2.

## Author contributions

JR designed, conducted, and analyzed experiments. TB, VN, and NC assisted in conducting experiments. TB contributed valuable discussion. JR prepared the figures and wrote the manuscript. JR, TB, and EB edited the manuscript. EB supervised the overall research.

### Conflict of interest statement

JR and EB are named on U.S. Patent #10,040,859: “Methods of treating hemophagocytic lymphohistiocytosis with an IL-33 receptor antibody.” The remaining authors declare that the research was conducted in the absence of any commercial or financial relationships that could be construed as a potential conflict of interest.
