# Supplementary material for: Disruption of IL-33 Signaling Limits Early CD8+ T Cell Effector Function Leading to Exhaustion in Murine Hemophagocytic Lymphohistiocytosis
Source: Front Immunol. 2018 Nov 20;9:2642. doi: 10.3389/fimmu.2018.02642 (PMC6256032; doi:10.3389/fimmu.2018.02642)

# Supplemental Data

**Disruption of IL-33 signaling limits early CD8+ T cell effector function leading to exhaustion in murine hemophagocytic lymphohistiocytosis**

Julia E. Rood^1-2^, Thomas Burn^1-2^, Vanessa Neal, Niansheng Chu^1^, Edward M. Behrens^1-2^

# Supplemental Figure Legends

## Figure S1. CD8^+^ T cell exhaustion in chronically-infected perforin-sufficient mice is largely independent of ST2.

WT and *Il1rl1^-/-^* mice (n=6/group) were infected with LCMV Cl-13 and assessed on day 15 p.i. An additional group of WT mice (n=4) was infected with LCMV Arm and analyzed on day 8 p.i. as a control. Data were analyzed by one-way ANOVA; significance of Tukey’s post-test comparing all groups is indicated. **(A)** Numbers of effector CD44^hi^CD62L^lo^ CD8^+^ T cells. **(B)** Numbers of gp33-specific CD8^+^ T cells. **(C-E)** Frequencies of gp33-tetramer^+^ CD8^+^ T cells expressing **(C)** PD-1, **(D)** 2B4, or **(E)** Ki-67. **(F)** Frequencies of Eomes^hi^ PD-1^hi^ cells among gp33-tetramer^+^ CD8^+^ T cells. **(G)** Frequencies (top row) and cytokine MFI (bottom row) of CD8^+^ T cells specifically producing cytokine or externalizing CD107a in response to *in vitro* gp33 peptide stimulation. Bars represent mean ± SEM. **(H)** Body weight of WT and *Il1rl1^-/-^* mice infected with LCMV Cl-13. Symbols represent mean ± SEM of 6 mice. Analyzed by two-way repeated measures ANOVA: there was a significant Interaction between Genotype and Time over the entire 30-day experiment (p<0.0001), but no significant interaction when comparing D15 to D30 weights.

## Figure S2. Reversal of CD8^+^ T cell exhaustion with α-PD-L1 treatment.

LCMV-infected *Prf1^-/-^Il1rl1^-/-^* mice were treated with 200 μg α-PD-L1 or isotype control antibodies every 3 days, beginning on day 15 p.i., and euthanized on day 30 p.i. for analysis. N=6 mice/group. Analyzed by Student’s two-tailed *t*-test. **(A-C)** Frequency of splenic CD8^+^ T cells producing **(A)** IFNγ or **(B)** TNFα, or **(C)** externalizing CD107a, in response to PMA/Ionomycin stimulation.

## Figure S3. Baseline immune cell subsets are similar between *Prf1^-/-^Il1rl1^-/-^* and *Prf1^-/-^* mice.

Baseline characteristics of splenocytes from uninfected *Prf1^-/-^* and *Prf1^-/-^Il1rl1^-/-^* mice. N=3 mice/group. **(A)** Total numbers of immune cell subsets. No significant effect of genotype in two-way ANOVA. **(B)** Representative flow plots of T cells. Numbers indicate frequency of gated population out of parent population. Top row, gated on live TCRβ^+^CD90.2^+^CD19^-^ cells. Middle row, gated on live TCRβ^+^CD90.2^+^CD8^+^ cells. Bottom row, gated on live TCRβ^+^CD90.2^+^CD4^+^ cells.

## Figure S4. Genetic deficiency of ST2 in LCMV-infected *Prf1^-/-^* mice phenocopies effects of α-ST2 antibody blockade.

*Prf1^-/-^Il1rl1^-/-^* mice were infected with LCMV and compared to LCMV-infected *Prf1^-/-^* mice receiving either isotype control antibody (*Prf1^-/-^* Control) or ST2-blocking antibody (*Prf1^-/-^* α-ST2) every other day, beginning on day 3 p.i. **(A)** Survival of LCMV-infected *Prf1^-/-^* Control (n=10), *Prf1^-/-^* α-ST2 (n=9), and untreated *Prf1^-/-^Il1rl1^-/-^* mice (n=14). Analyzed by log-rank (Mantel-Cox) test. **(B)** Body weight. Symbols represent mean ± SEM of 9-14 mice. Pairwise comparisons of groups were analyzed by linear mixed-effects model. **(C-F)** Analysis of splenocytes 8 days p.i. N=4 mice/group. Analyzed by one-way ANOVA; significance of Tukey’s post-test comparing all groups is indicated. **(C)** Serum IFNγ level. **(D)** Numbers of gp33-specific CD8^+^ T cells. **(E)** Frequencies and **(F)** median IFNγ fluorescence intensity of IFNγ^+^ gp33-specific CD8^+^ T cells. **(G-I)** Analysis of splenocytes from *Prf1^-/-^Il1rl1^-/-^* mice 31 days p.i. WT mice infected with LCMV Arm and analyzed on day 15 p.i. served as an LCMV-immune control. N=5 mice/group. Analyzed by Student’s two-tailed *t*-test. **(G)** MFI of PD-1 on gp33 tetramer^+^ CD8^+^ T cells. **(H)** Frequencies and **(I)** median IFNγ fluorescence intensity of IFNγ^+^ gp33-specific CD8^+^ T cells.

# Supplemental Figures

## Figure S1


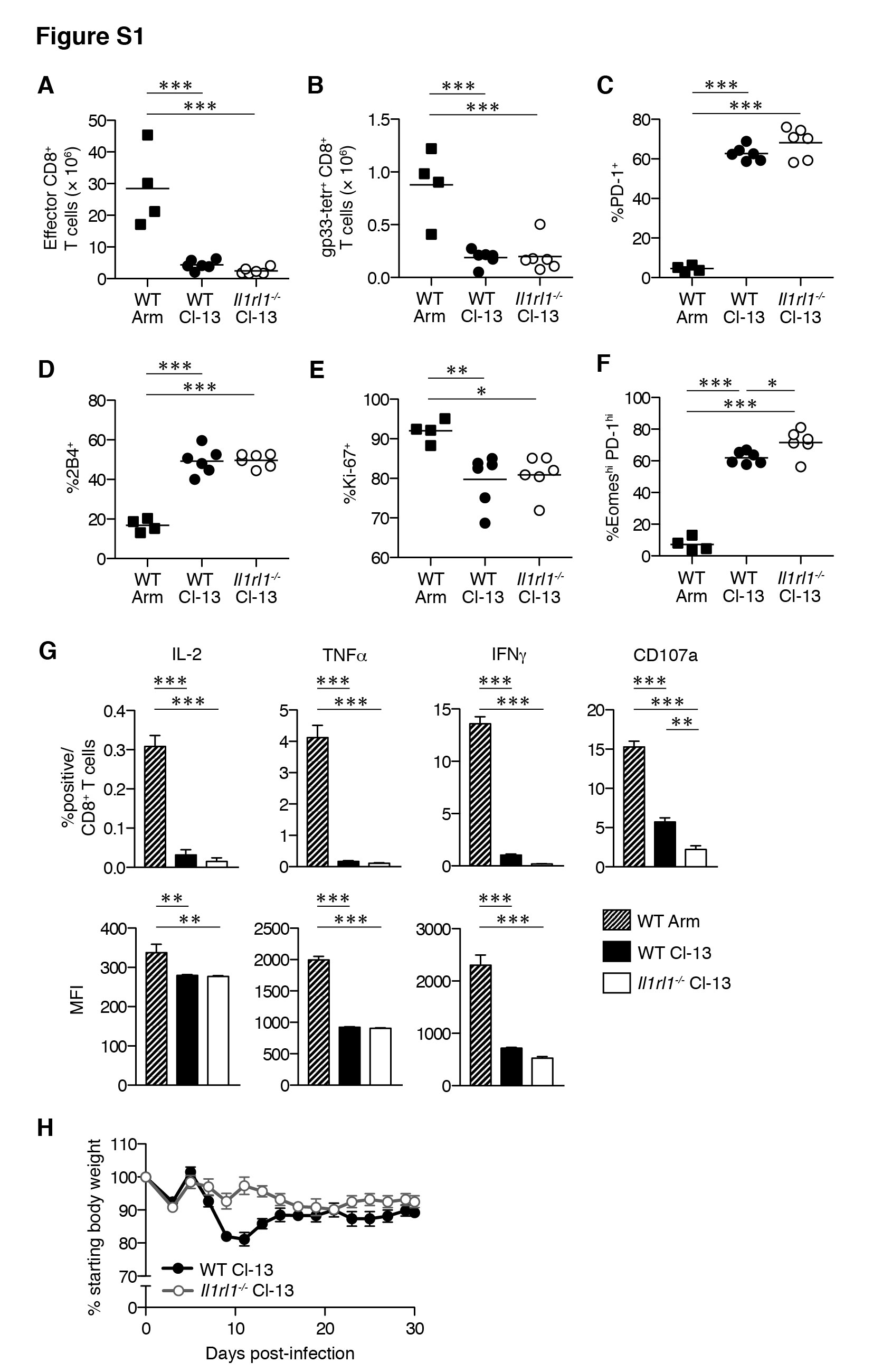


## Figure S2


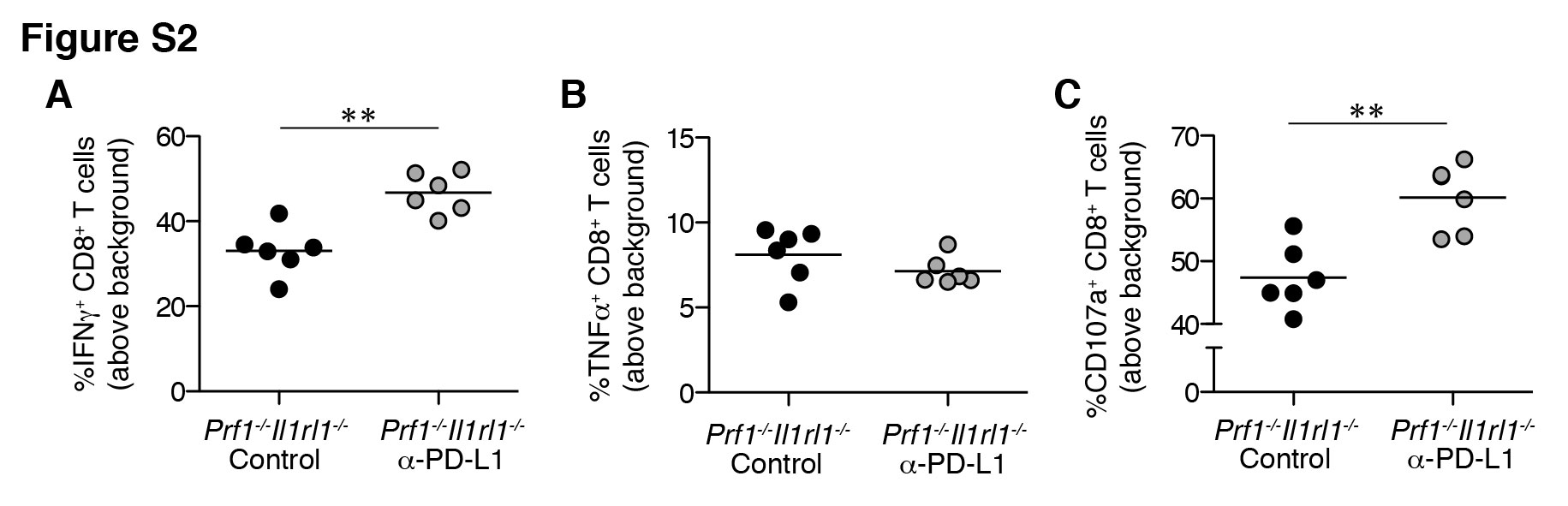


## Figure S3


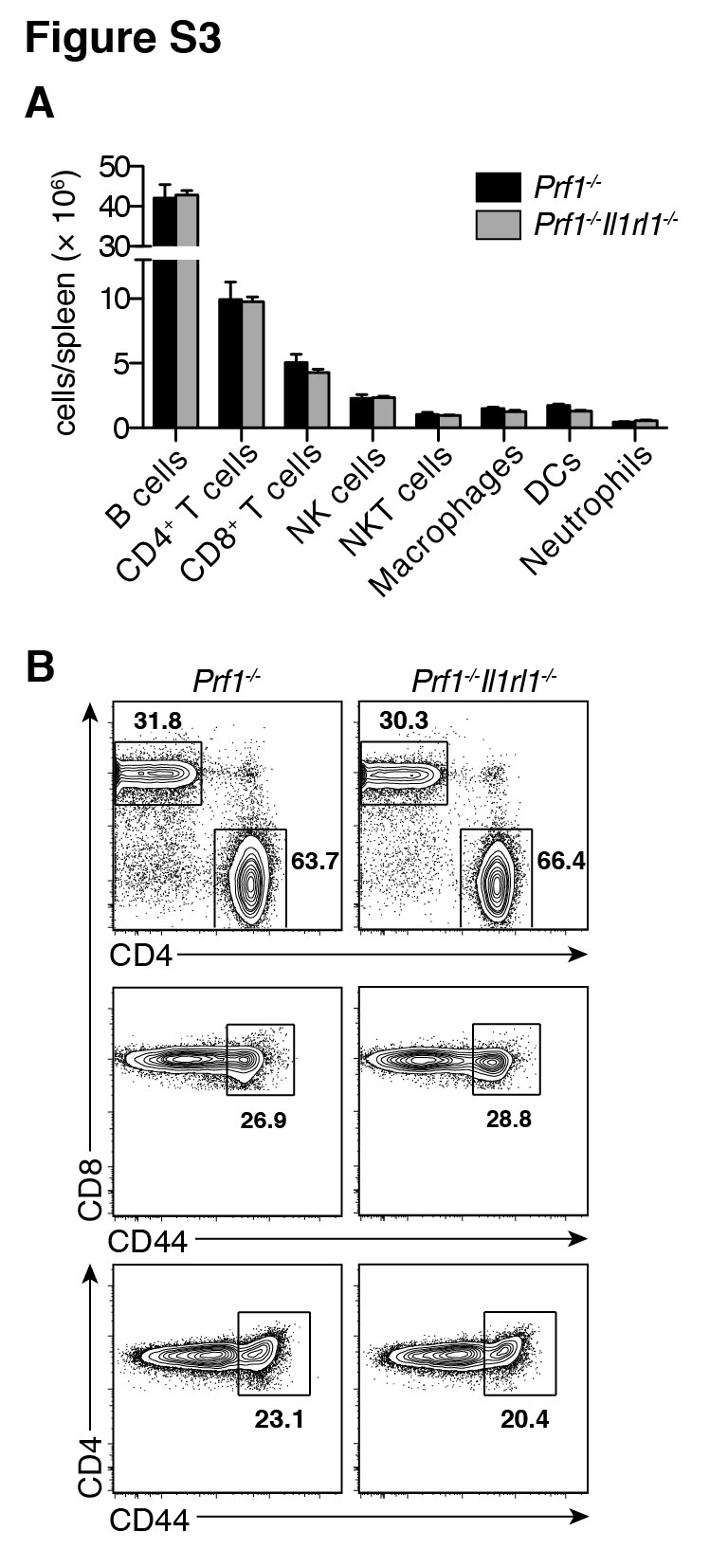


## Figure S4


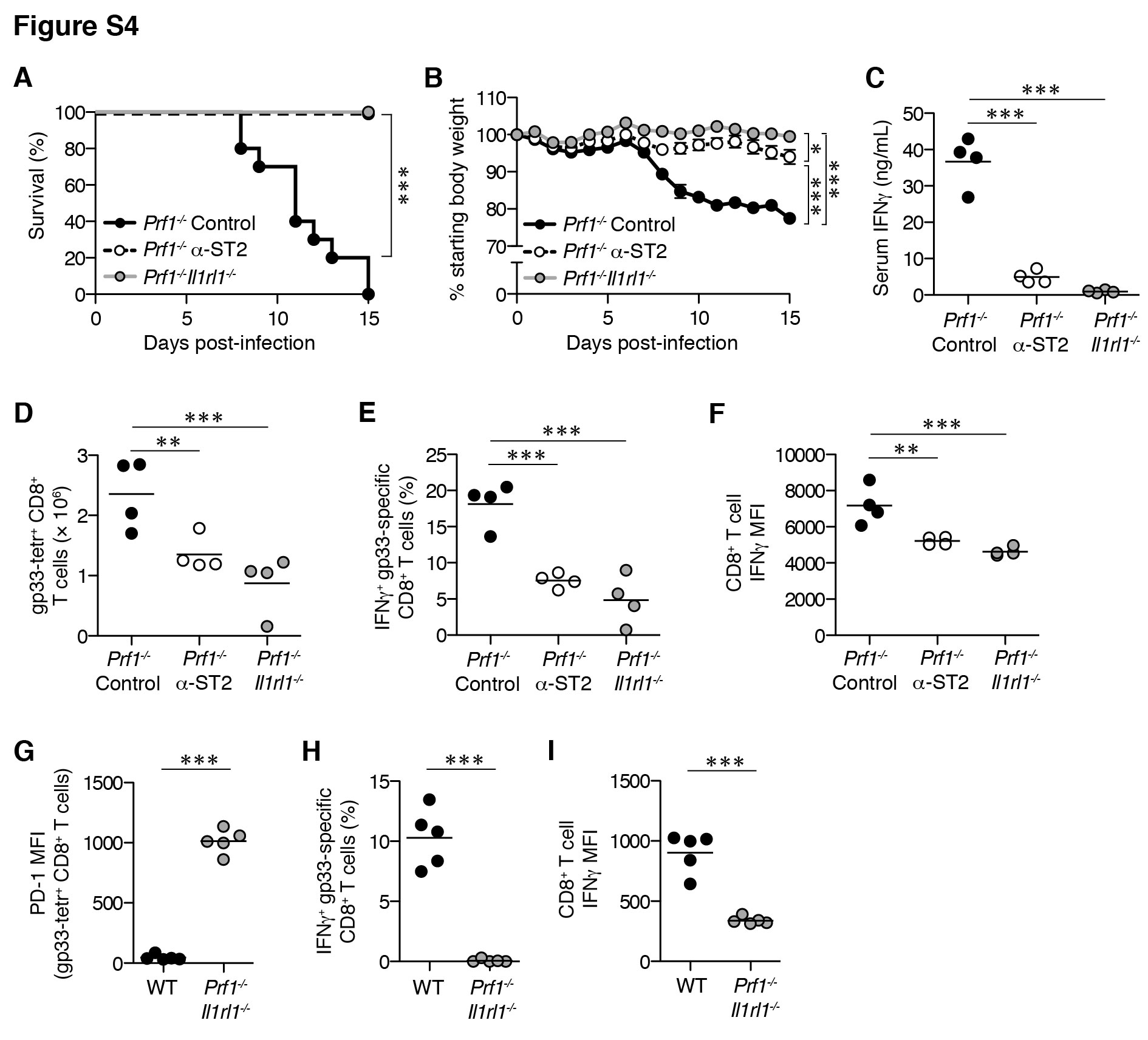

Supplement: Supplementary file 1 [file Data_Sheet_1.docx]
